# ATP-Binding Cassette Subfamily a Member 2 Is a Functional Receptor for *Bacillus thuringiensis* Cry2A Toxins in *Bombyx mori*, But Not for Cry1A, Cry1C, Cry1D, Cry1F, or Cry9A Toxins

**DOI:** 10.3390/toxins12020104

**Published:** 2020-02-06

**Authors:** Xiaoyi Li, Kazuhisa Miyamoto, Yoko Takasu, Sanae Wada, Tetsuya Iizuka, Satomi Adegawa, Ryoichi Sato, Kenji Watanabe

**Affiliations:** 1 Graduate School of Bio-Applications and Systems Engineering, Tokyo University of Agriculture and Technology, Naka 2-24-16, Koganei, Tokyo 184-8588, Japan; s159217x@st.go.tuat.ac.jp (X.L.); s174092v@st.go.tuat.ac.jp (S.A.); 2 Institute of Agrobiological Sciences, NARO, 1-2 Ohwashi, Tsukuba, Ibaraki 305-8634, Japan; miyamoto401606@gmail.com (K.M.); takasu@affrc.go.jp (Y.T.); sunny@affrc.go.jp (S.W.); tiizuka@affrc.go.jp (T.I.)

**Keywords:** Cry2Ab toxin, *Bombyx mori*, ATP-binding cassette subfamily a member 2 (ABCA2), genome editing, transcription activator-like effector-nucleases (TALENs), HEK293T cell, functional receptor

## Abstract

Cry toxins are insecticidal proteins produced by *Bacillus thuringiensis* (Bt). They are used commercially to control insect pests since they are very active in specific insects and are harmless to the environment and human health. The gene encoding ATP-binding cassette subfamily A member 2 (ABCA2) was identified in an analysis of Cry2A toxin resistance genes. However, we do not have direct evidence for the role of ABCA2 for Cry2A toxins or why Cry2A toxin resistance does not cross to other Cry toxins. Therefore, we performed two experiments. First, we edited the *ABCA2* sequence in *Bombyx mori* using transcription activator-like effector-nucleases (TALENs) and confirmed the susceptibility-determining ability in a diet overlay bioassay. Strains with C-terminal half-deleted BmABCA2 showed strong and specific resistance to Cry2A toxins; even strains carrying a deletion of 1 to 3 amino acids showed resistance. However, the C-terminal half-deleted strains did not show cross-resistance to other toxins. Second, we conducted a cell swelling assay and confirmed the specific ability of BmABCA2 to Cry2A toxins in HEK239T cells. Those demonstrated that BmABCA2 is a functional receptor for Cry2A toxins and that BmABCA2 deficiency-dependent Cry2A resistance does not confer cross-resistance to Cry1A, Cry1Ca, Cry1Da, Cry1Fa or Cry9Aa toxins.

## 1. Introduction

Cry toxins are insecticidal crystal proteins and pore-forming toxins produced by *Bacillus thuringiensis* (Bt) [1,2]. Proteases activate Cry toxins in the midgut of the host insect; the toxins then interact with specific receptors on the columnar cell membrane [3]. This interaction drives the toxins to insert partial structures into the membrane, forming ion channels [4]. A cation influx triggers the influx of water [5], resulting in cell swelling and lysis [2,6]. Given their strong toxicity in specific species and inability to harm the environment and human health, Cry toxins are used widely in pest control [7]. As a gene source, Bt toxin genes have been used efficiently to make transgenic crops (Bt crops) that resist pests [8]. However, resistant insect strains have been found in these crops [9].

To delay the selection and evolution of resistance in exposed insect populations, current commercial insecticidal systems combine two or more Cry toxins that bind to different receptors in the target pests [10]. Nevertheless, in traditional insecticidal systems that use a single Cry toxin, the generation of resistant insects and cross-resistance to other Cry toxins are still problems, reducing the value of commercial Bt crops. Regarding cross-resistance, in *Ostrinia nubilalis* and *Spodoptera frugiperda*, Cry1Ab, Cry1Ac, and Cry1Fa compete for the same binding sites with high affinity; however, Cry2Ab does not compete for the binding sites of Cry1 proteins [10]. Heterologous competition binding assays in *Helicoverpa armigera* and *Helicoverpa zea* midguts showed a common binding site for three Cry2A toxins (Cry2Aa, Cry2Ab, and Cry2Ae), but this binding site was not shared with Cry1Ac [11]. Cry 1Ab-resistant *Pectinophora gossypiella* was also reported to have strong cross-resistance to Cry1Aa, but little or no cross-resistance to Cry1Ca, Cry1Da, Cry2Aa, and Cry9Aa [12]. In the diamondback moth (*Plutella xylostella*), the Cry1C-resistant strain had strong cross-resistance to Cry1Ab, Cry1Ac, and Cry1F, but low cross-resistance to Cry1Aa and no cross-resistance to Cry2Aa [13]. Therefore, it is necessary to clarify the reason for cross-resistance or the receptors used by each Cry toxin to devise new strategies to defeat cross-resistance.

ATP-binding cassette (ABC) transporters, a class of transmembrane proteins, are found widely in organisms and are involved in Bt toxin activities [14]. Many studies seeking to identify functional receptors of target Cry toxins have focused on ABC transporters [15]. When BmABCC2 was expressed in *S. frugiperda* (Sf9) cells and *Drosophila* tissues that were originally insensitive, they became sensitive to Cry1 toxins, and when BmABCC2 was expressed in *Xenopus* oocytes the Cry toxins made pores in the membrane and cations flowed into the cells through these pores [16,17,18]. These results indicate that BmABCC2 is a receptor for Cry1A toxins. Using heterologous expression in Sf9 cells, ABCB1 was found to be a functional Cry3Aa receptor [19]. In comparison, an *H*. *armigera* strain with 6000-fold resistance to Cry2Ab [15] had a mutation in ABC subfamily A member 2 (HaABCA2), suggesting that ABCA2 is linked to Cry2Ab resistance [20]. This was confirmed using CRISPR/Cas9-mediated genome editing, leading to the conclusion that HaABCA2 determines the susceptibility of *H*. *armigera* to Cry2Aa and Cry2Ab [21]. The knockout of ABCA2 using CRISPR/Cas9 conferred resistance to Cry2Ab on *Trichoplusia ni* [22]. Furthermore, in Cry2Ab-resistant *P*. *gossypiella* strains, PgABCA2 was disrupted in several ways, indicating that the Cry2Ab-resistance of *P*. *gossypiella* is associated with an ABCA2 deficiency [23]. Therefore, ABCA2s seem to cause Cry2 resistance in most insects. In addition, since ABCC2 and ABCB1 function as receptors for Cry1A and Cry3A toxins, respectively, ABCA2s likely function as receptors for Cry2A toxins. However, to confirm whether ABCAs are really functional receptors for Cry2 toxins, it is necessary to show the receptor function of ABCAs using a heterologous expression system.

In this study, we used transcription activator-like effector nucleases (TALEN) to edit the genome at the *BmABCA2* locus and created mutant strains of *Bombyx mori* as a model system to clarify susceptibility determining activity. Then, we performed Cry2A toxin-contaminated leaf disc feeding assays using these mutant strains to determine whether BmABCA2 is really a susceptibility determinant for Cry2A toxins in *B*. *mori*. Using HEK293T cells expressing BmABCA2, we conducted cell swelling assays to demonstrate the Cry2A toxin-specific receptor function of BmABCA2.

## 2. Results

### 2.1. Creation of Silkworm Strains with C-Terminal Half-Deleted BmABCA2s and Mutants with C-Terminal Deletions in TM7 by TALEN-Mediated Mutagenesis

**Figure 1:**
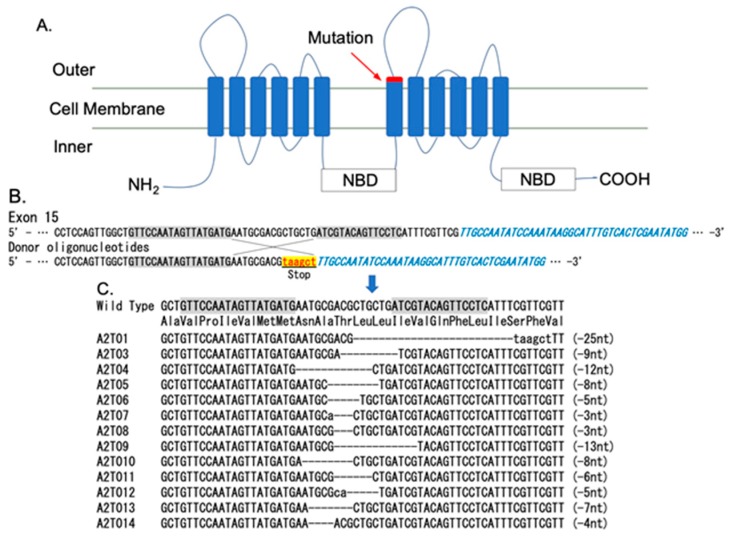
TALEN-induced mutations generated at the C-terminus of TM7 in BmABCA2. (**A**) Schematic structure of BmABCA2 and the mutation sites created with the TALEN system in the wild-type (Ringetsu) *B. mori* strain. The transmembrane topology of BmABCA2 was predicted by Phobius (http://phobius.sbc.su.se/). The approximate position of the mutation is indicated by the red rectangle located at the C-terminus of TM7 in BmABCA2. (**B**) The TALEN-binding site to TM7 in exon 15 used to introduce an insertion/deletion mutation in BmABCA2 and donor oligonucleotides for HDR. (**C**) The detected G_1_ mutant alleles of the mutant strains. A2T01 was obtained as a result of HDR, while A2T03–A2T14 were obtained as the result of NHEJ.

First, 200 eggs were injected individually with TALEN mRNAs and a donor oligonucleotide prepared to mutate the C-terminus of transmembrane domain 7 (TM7) in exon 15 (Figure 1A,B). The 14 neonates that hatched were allowed to develop (G_0_). Two female moths survived and were crossed singly with the wild-type strain to produce the next generation (G_1_). The G_1_ larvae were allowed to develop into adults. Genomic DNA samples of individual G_1_ moths were prepared, and insertion/deletion mutations at TM7 in exon 15 of *BmABCA2* were identified. One mutant allele (named A2T01) was presumably derived from homology-directed repair (HDR), and 12 mutant alleles (named A2T03–A2T14) obtained via non-homologous end joining (NHEJ) were detected (Figure 1C). The mutations in A2T01, A2T05, A2T06, A2T09, A2T10, A2T12, A2T13, and A2T14 led to truncation of the C-terminus of TM7 in BmABCA2. The mutations in A2T03, A2T04, A2T07, A2T08, and A2T11 were predicted to cause 3, 4, 1, 1, and 2 amino-acid deletions from the C-terminus of TM7, respectively.

To construct homozygous strains, heterozygous G_1_ strains were selected after confirming the PCR products with direct sequencing and were mated with individuals of the same genotype. The homozygous individuals of the next generation (G_2_) were screened by genotyping, resulting in the establishment of three strains with C-terminal half-deleted BmABCA2 (A2T01, A2T06, and A2T14) and three strains (A2T03, A2T08, and A2T11) with amino acids deleted from the C-terminus of TM7 (Figure S1).

### 2.2. BmABCA2 Activity against Cry2A Toxins

**Figure 2:**
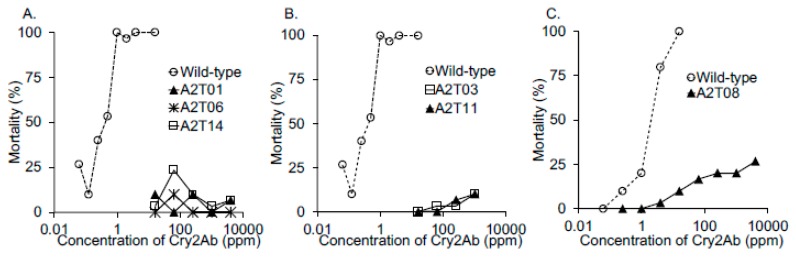
Toxin feeding assay to evaluate the susceptibility of the larvae with mutations in BmABCA2 to Cry2Ab. (**A**) Evaluation of strains with C-terminal half-deleted BmABCA2s (A2T01, A2T06, and A2T14; indicated in Figure 1C. The wild-type strain (Ringetsu), which is susceptible to Cry2A toxins, was used as a control. (**B**,**C**) The susceptibility of strains with BmABCA2s carrying 1–3 amino acid deletions at the C-terminus of TM7 [A2T03, A2T11 (**B**), and A2T08 (**C**); indicated in Figure 1A,C] to Cry2Ab. The wild-type strain was used as a control. Cry2Ab was spread on the leaf disk at 10 μL/cm^2^. The toxin feeding assays were performed as described in the Materials and Methods.

To test the susceptibility of the homozygous BmABCA2 mutant strains to Cry2Ab, larvae were reared on leaf disks contaminated with Cry2Ab for 2 days, and then on leaf disks without toxin for an additional 2 days. Larvae of all of the strains with C-terminal half-deleted BmABCA2s (A2T01, A2T06, and A2T14; Figure 1) showed strong resistance to Cry2Ab (Figure 2A). The median lethal dose (LC_50_) of Cry2Ab on A2T14 was >9990-fold higher than that of the wild-type strain (Table 1). Surprisingly, the larvae of strains with BmABCA2s in which 1–3 amino acids had been deleted from the C-terminus of TM7 (A2T03, A2T11, and A2T08; Figure S1), were also resistant to Cry2Ab (Figure 2B,C). However, in association with the Cry2Ab concentration, those strains were slightly susceptible to Cry2Ab, i.e., the resistance of those strains was not as high as that of strains with C-terminal half-deleted BmABCA2s.

**Figure 3:**
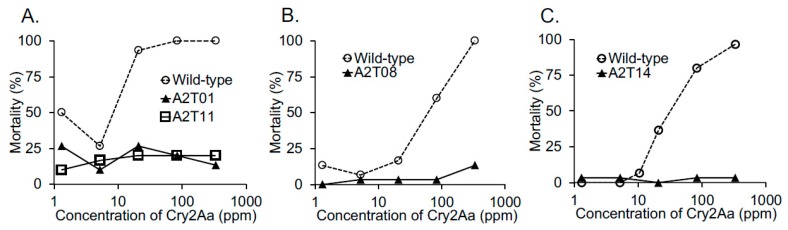
Toxin feeding assay to evaluate the susceptibility of larvae with mutations in BmABCA2 to Cry2Aa. Strains with C-terminal half-deleted BmABCA2s [A2T01 (**A**) and A2T14 (**C**)] and strains with BmABCA2s carrying 1 or 2 amino acid deletions at the C-terminal end of TM7 [see Figure 1A,C; A2T08 (**B**) and A2T11 (**A**)] were evaluated using the toxin feeding assay as described in Figure 2 and the Materials and Methods. The wild-type strain (Ringetsu) was used as a control.

The susceptibility of *B*. *mori* strains carrying mutant BmABCA2 to Cry2Aa was also investigated. All of the strains with C-terminal half-deleted BmABCA2s (A2T01 and A2T14) and the strains with BmABCA2s in which 1 or 2 amino acids were deleted from the C-terminus of TM7 (A2T08 and A2T11) were resistant to Cry2Aa (Figure 3). With increasing Cry2Aa concentrations, A2T08 showed slight susceptibility to Cry2Aa (Figure 3B).

**Figure 4:**
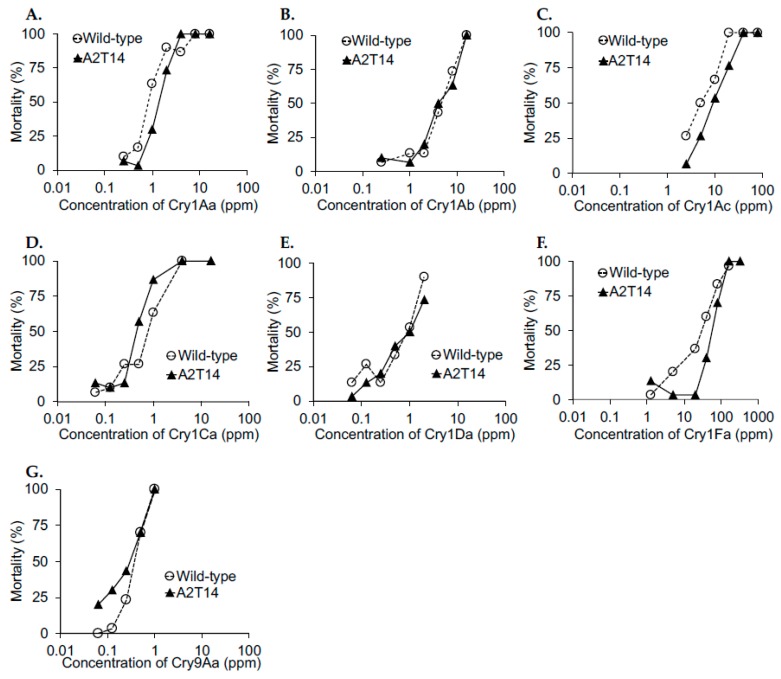
Toxin feeding assay to evaluate the susceptibility of the *B*. *mori* strain with a C-terminal half-deleted BmABCA2 (A2T14) to several Cry toxins. The strain with C-terminal half-deleted BmABCA2 (A2T14) and the wild-type strain (Ringetsu) were fed leaf disks contaminated with Cry1Aa (**A**), Cry1Ab (**B**), Cry1Ac (**C**), Cry1Ca (**D**), Cry1Da (**E**), Cry1Fa (**F**), or Cry9Aa (**G**) as described in the Materials and Methods.

The susceptibility of a *B*. *mori* strain with C-terminal half-deleted BmABCA2s (A2T14) to Cry1A, Cry1Ca, Cry1Da, Cry1Fa, and Cry9Aa was investigated further. We found that the susceptibility of strain A2T14 to Cry1Aa, Cry1Ab, Cry1Ac, Cry1Ca, Cry1Da, Cry1Fa, and Cry9Aa was similar to that of the wild-type strain (Figure 4). Moreover, the LC_50_ with the 95% confidence interval of each toxin was calculated (Table 1). The LC_50_ of every toxin did not differ between the wild-type and A2T14 strains, but A2T14 strains had no or very limited resistance to Cry1Aa (<2-fold), Cry1Ac (<5-fold) and Cry1Fa (<3-fold), indicating that the knockout of BmABCA2 did not affect susceptibility to Cry1A, Cry1Ca, Cry1Da, Cry1Fa, or Cry9Aa.

### 2.3. BmABCA2-Dependent Cry2A Toxins Induce Cell Swelling

**Figure 5:**
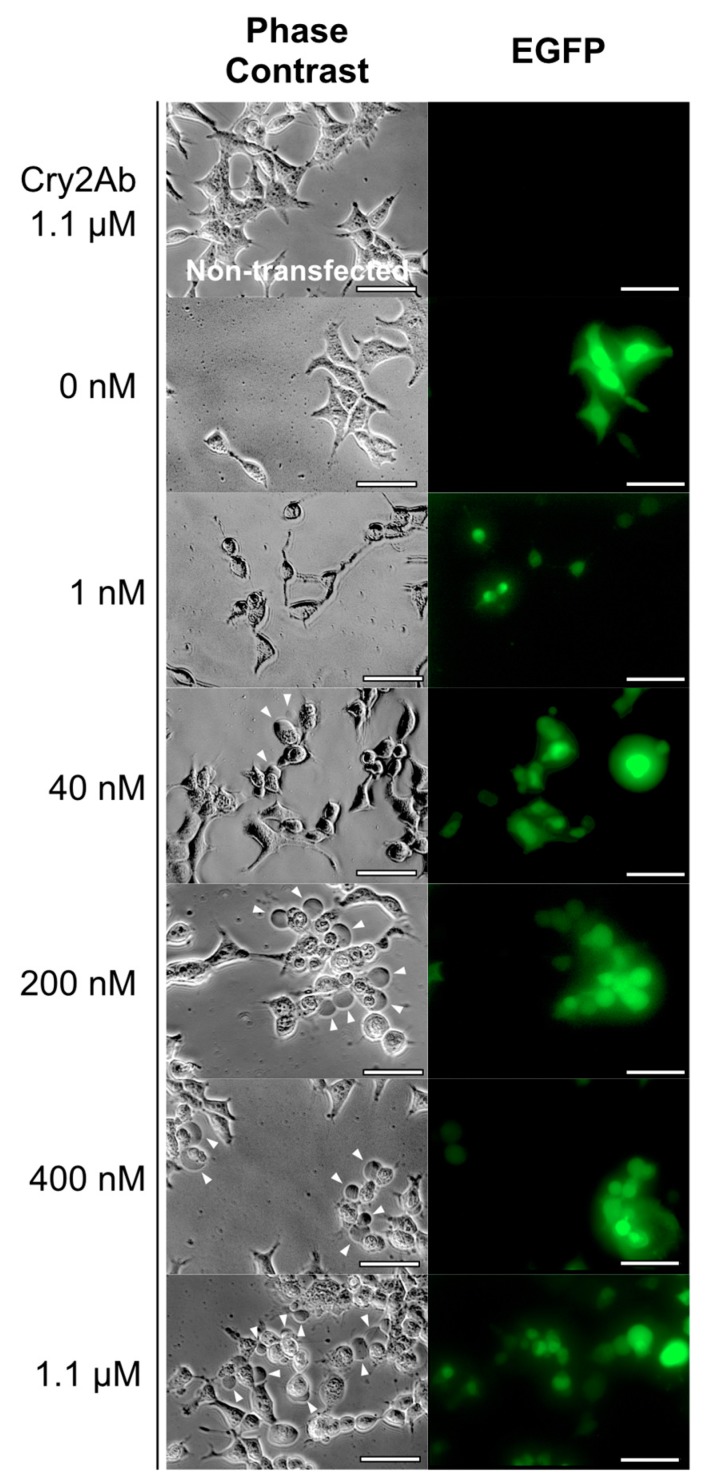
Cell swelling assay against Cry2Ab using BmABCA2-expressing HEK292T cells. The cells were attached to coverslips set on the six plates. BmABCA2 was transiently expressed on the surface of HEK293T cells via the transfection of an expression vector with attached EGFP. The coverslips were set on the wells of glass slides filled with the Cry2Ab test solutions, incubated for 1 h, and observed under a microscope. Arrowheads indicate swollen cells. (Scale bar = 50 μm).

To examine whether BmABCA2 is a functional Cry2A toxin receptor, we used cell swelling assays. Enhanced green fluorescent protein (EGFP) cDNA was equipped with BmABCA2 cDNA in the same vector and transiently expressed in HEK293T cells showing green fluorescence on transfection. Cry2Ab was administered to those transient expression cells. Only the EGFP-positive cells swelled when they were treated with more than 40 nM Cry2Ab (Figure 5). The cells swelled in a Cry2Ab toxin concentration-dependent manner (Figure 5). By contrast, even 1.1 µM Cry2Ab did not induce swelling in cells not transfected with the BmABCA2 expression vector (Figure 5). Furthermore, cells that were transfected with the BmABCA2 expression vector did not swell when they were incubated in buffer lacking Cry2Ab.

**Figure 6:**
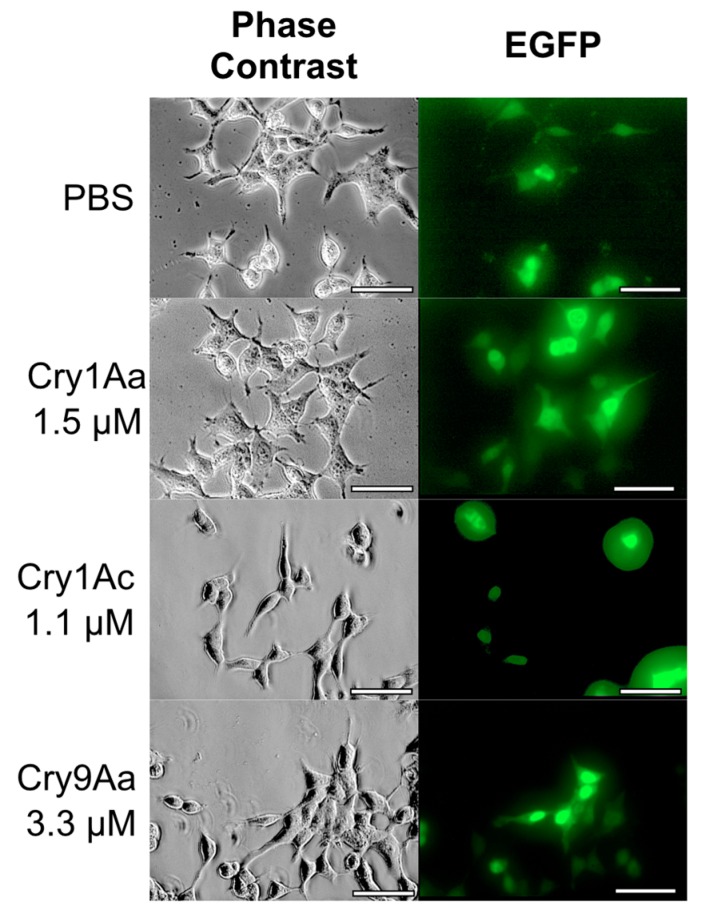
Cell swelling assay against Cry1A toxins and Cry9Aa using BmABCA2-expressing HEK293T cells. The cells were attached to coverslips set on the six plates. BmABCA2 was transiently expressed on the surface of HEK293T cells via the transfection of an expression vector with attached EGFP. Then, the cells were incubated for 1 h with Cry toxins on glass slides and observed under a microscope, as described in Figure 5. (Scale bar = 50 μm).

To examine whether BmABCA2 acts as a functional receptor for other Cry toxins, BmABCA2-expressing cells were treated with Cry1Aa, Cry1Ac, and Cry9Aa. However, no cells were swollen after treatment with up to 1.5 µM Cry1Aa, 1.1 µM Cry1Ac, and 3.3 µM Cry9Aa (Figure 6).

**Figure 7:**
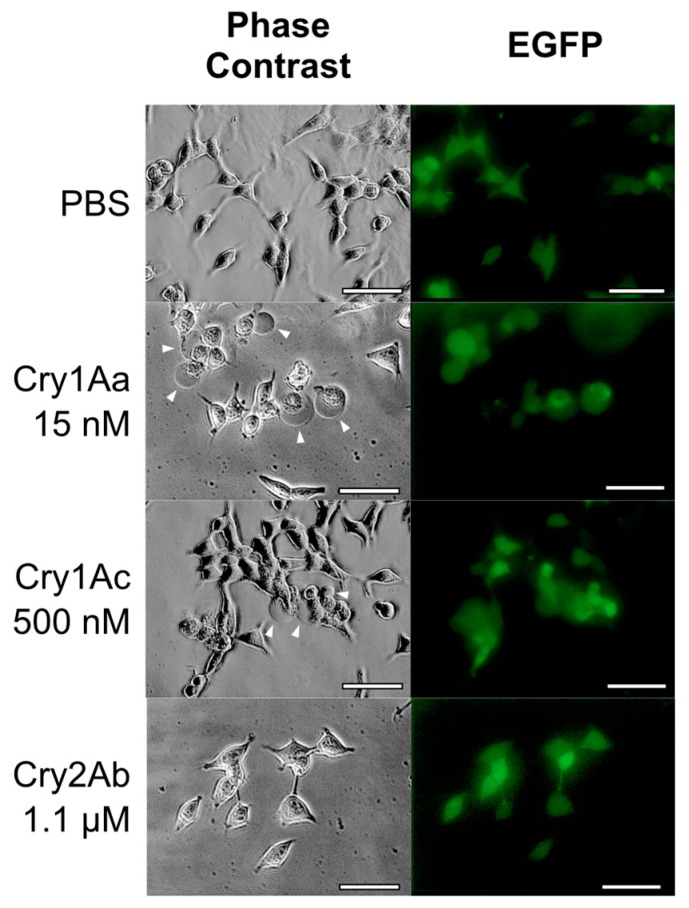
Cell swelling assay against Cry1A toxins and Cry2Ab using BmABCC2-expressing HEK293T cells. The cells were attached to coverslips set on the six plates. BmABCC2 co-expressed with EGFP was transiently expressed on the surface of HEK293T cells via transfection of the expression vector. Then, the cells were incubated for 1 h with Cry toxins on glass slides and observed under a microscope, as described in Figure 5. Arrowheads indicate swollen cells. (Scale bar = 50 μm).

To clarify whether the BmABCC2 receptor for Cry1A toxins can function as a Cry2Ab receptor, BmABCC2-expressing cells were administered Cry2Ab. However, no cells swelled with up to 1.1 µM Cry2Ab (Figure 7). By contrast, Cry 1Aa induced swelling in BmABCC2-expressing cells at 15 nM, indicating that the level of BmABCC2 expression was sufficient to assess Cry2Ab receptor function (Figure 7). Cry1Ac induced swelling of the BmABCC2-expressing cells at 500 nM (Figure 7).

## 3. Discussion

Toxicity tests of the strains with C-terminal half-deleted BmABCA2 (A2T01, A2T06, and A2T14) showed that they were highly resistant to Cry2Ab (Figure 2), indicating that BmABCA2 plays an essential role in determining the susceptibility of *B*. *mori* to Cry2Ab. ABCA2 was first suggested to be linked to Cry2Ab resistance in *H*. *armigera* [20]. This was confirmed by generating an ABCA2 knockout strain via CRISPR/Cas9 mutagenesis in which the resistance of *H*. *armigera* and *T*. *ni* larvae to Cry2Ab was linked to defects in ABCA2 [21,22], indicating that ABCA2 plays an important role in the mode of action of Cry2A toxins. Although the resistance was slightly lower than that of the C-terminal half-deleted strains (Figure 2B,C), toxicity testing of the strains with BmABCA2s that had 1–3 amino acids deleted from the C-terminal end of TM7 (A2T03, A2T08, and A2T11) showed that they were also resistant to Cry2Ab (Figure 2B,C). Thus, BmABCA2 also plays an essential role in determining the susceptibility to Cry2Ab in *B*. *mori*. In *B*. *mori*, even the partial deletion and alanine replacement of several amino acid residues at the N-terminus of extracellular loop 4 (ECL4) decreased the susceptibility-conferring activity of BmABCC2 to Cry1Aa [17]. This suggests that ECL4 of BmABCC2 is part of the site that interacts with Cry1Aa. ECL4 of BmABCA2 might also play a role in the interaction with Cry2Ab (Figure 1A), and the 1–3 amino acids that were deleted from the C-terminal end of TM7 might affect the interaction of BmABCA2 with Cry2Ab by changing the three-dimensional structure of the binding epitopes.

It is still not known how ABCA2 is involved in the susceptibility of larvae to Cry2A toxins. ABCA2 is a membrane protein and the ABC transporter ABCC2 is a functional Cry1A toxin receptor [5,16,18,24,25]. Therefore, we examined whether BmABCA2 transiently expressed in HEK293T cells (Figure 5) could function as a Cry2Ab receptor and whether HEK293T cells would swell like the columnar cells in a Cry toxin-intoxicated midgut. The BmABCA2-expressing cells started swelling on exposure to 40 nM Cry2Ab (Figure 5). When BmABCC2, a highly functional receptor for a single Cry1Aa molecule [16], was expressed in HEK293T cells using a very similar expression system, the HEK293T cells started swelling in response to 1 nM Cry1Aa [26]. By contrast, when BmABCC3, a less functional receptor for a single Cry1Aa molecule, was expressed in HEK293T cells, the cells started to swell in response to 100 nM Cry1Aa [26]. Based on our cell swelling assays using HEK293T cells, BmABCA2 has sufficient functional receptor activity for Cry2Ab. This is consistent with the strong resistance to Cry2Ab that was generated by the TALEN-induced BmABCA2 mutation (Figure 2).

In *H*. *armigera*, an HaABCA2 knockout strain showed resistance to Cry2Ab, but not to Cry1Ac [21], suggesting that this receptor is highly tuned to Cry2A toxins. Regarding Cry1Aa, the BmABCC2 binding site was thought to be the pocket made by loops 2 and 3 [27]. However, we could not find any amino acids near loops 2 and 3 that were conserved in Cry1Aa and Cry2Ab. This might explain the receptor specificity difference between Cry1Aa and Cry2Ab. The BmABCA2 knockout strains were susceptible to Cry1Aa (Figure 4). In addition, the BmABCA2 knockout strains were susceptible to all of the Cry1 and Cry9 toxins tested (Figure 4). This implies that Cry2A toxin-resistant insects lack cross-resistance to Cry1Ca, Cry1Da, Cry1Fa, or Cry9Aa. Actually, a Cry1Ca-resistant diamondback moth was reported to lack cross-resistance to Cry2Aa [13]. Furthermore, BmABCA2-expressing HEK293T cells were susceptible to Cry2Ab, but not to Cry1A or Cry9A toxins (Figure 5 and Figure 6). Therefore, our results suggest that the specificity of BmABCA2 as a Cry toxin receptor is narrowly tuned to Cry2A toxins. By contrast, BmABCC2-expressing HEK293T cells were susceptible to Cry1A toxins, but not to Cry2Ab (Figure 7). This also suggests that ABC transporters are highly tuned to a narrow group of Cry toxins.

## 4. Materials and Methods

### 4.1. Silkworm Strains and Rearing

The wild-type silkworm strain was distributed from the Genetics Resources Center, National Agriculture and Food Research Organization (NARO) and was reared on mulberry leaves or artificial diet (Nihon Nosan Kogyo, Yokohama, Japan) at 25 °C.

### 4.2. DNA Target Site Selection and Preparation of TALEN mRNA

The DNA target site was selected in the fifteenth exon of the BmABCA2 (KP219767) gene that encodes the extracellular region between the 7th and 8th transmembrane regions. Two TALEN half sites were designed, as shown in Figure 1. TALEN-encoding genes were constructed by Golden Gate assembly, as described previously [28]. To prepare mRNAs for microinjection, TALEN-encoding plasmids were linearized by *Xba*I (TaKaRa Bio, Kusatsu, Japan) and transcribed using an mMESSAGE mMACHINE T7 transcription kit (Thermo Fisher Scientific, Waltham, MA, USA) according to the protocols of manufacturer.

### 4.3. Egg Microinjection

The poly(A)-tailed TALEN mRNAs (0.2 μg/μL) were dissolved in injection buffer (5 mM KCl, 0.5 mM phosphate buffer, pH 7.0) together with donor oligonucleotides (0.2–0.4 μg/μL), as described previously [29], and injected to the silkworm eggs at the syncytial preblastodermal stage [30]. The embryos were incubated at 25 °C in a humidified atmosphere.

### 4.4. Identification of BmABCA2 Mutation Induced by TALENs

To extract genomic DNA, a leg of each G_1_ moth was homogenized in 50 μL of DNAzol^®^Direct (Molecular Research Center, Cincinnati, OH, USA). After 10 min of incubation at room temperature, the homogenate was mixed vigorously and separated by centrifugation. The supernatant containing genomic DNA was used as a template for PCR. The target region of the BmABCA2 gene was amplified using a specific primer set (forward: 5′-GTGTCAGGAGCAAGTCTGGTC-3′, reverse: 5′-AGACGTGTTAAATATCTCGTCTCG-3′). Direct sequencing of the PCR products was performed using the reverse primer as a sequencing primer. Mutations induced by TALENs were identified according to the sequencing results.

### 4.5. Cry Toxins Preparation

The DNAs of Cry2Ab (AAA22342), and Cry1Fa (AAA22348) genes which were optimized for expression of heterologous proteins in *Escherichia coli* was synthesized by Strings DNA Fragments service (Thermo Fisher Scientific) and subcloned into between *Bam*HI and *Xho*I sites of pGEX6P-3 (GE healthcare lifesciences, Amersham, UK) using In-fusion HD Cloning kit (TaKaRa Bio, Kusatsu, Japan). The DNA clones were used to produce the Cry2Ab, and Cry1Fa toxins. For the production of the Cry1Aa, Cry1Ab, Cry1Ac, Cry1Da, and Cry9Aa toxins, the genes of these toxins were subcloned into pGEX4T-3 and then *E. coli* cells were transformed with those as described previously [16]. The transformed cells were cultured in LB liquid medium with ampicillin at 37 °C and gene expression was induced by isopropyl thio-b-d-galactoside. The inclusion bodies were harvested and washed as describe as previously [31]. Inclusion bodies of Cry2Aa toxin was prepared as described elsewhere [32]. The Cry1Ca toxin was produced by a *B. thuringiensis* recombinant stain, and inclusion bodies of Cry1Ca were washed as described elsewhere [33]. The Cry1Aa, Cry1Ac, and Cry9Aa were solubilized and activated as described previously [27]. The inclusion bodies of Cry2Ab was activated with a High-Performance Liquid Chromatography method (HPLC) that the Cry2Ab was solubilized as same as described above. After the solution, the pro-toxin of Cry2Ab was dialyzed with 20 mM Tris-HCl pH 9 and passed through a 0.45 µM filter (Millipore Millex-HP Hydrophilic PVDF, Millipore, Burlington, MA, USA) to remove bacteria. Then, the pro-toxin was applied to an HPLC system equipped with a Shodex IEC DEAE-825 column (0.8 × 7–5cm, Showa Denko Co.) and equilibrated with 250 mM Tris-HCl, pH 9. The pro-toxin was bound with the column, and non-absorbent pellets were washing by 100 mM Tris-HCl pH 9. Then, the bound pro-toxin was treated with 0.0625 mg/mL Trypsin (Sigma-Aldrich, St. Louis, MO, USA) for 40 min at 37 °C. The treatment Cry2Ab was eluted by Elix water 5 min later, with a linear gradient of 0~250 mM Tris-HCl pH 9 buffer eluted over 55 min with a 0.5 mL/min flow rate, and the activated toxin was dialyzed by phosphate-buffered saline (PBS, 137 mM sodium chloride, 2.7 mM potassium chloride, 10 mM sodium phosphate dibasic, 1.8 mM potassium dihydrogen phosphate, pH 7.4) for cell swelling assay.

### 4.6. Diet Overlay Bioassays

Susceptibility to Cry toxins of the BmABCA2 genome edited mutants and wild-type strains were evaluated with diet overlay bioassays. To make leaf disks, sixth open leaves from the top of each branch of the mulberry leaves were picked up. Cry toxins solutions were diluted with Silwet^®^ L-77 (Momentive Performance Materials, Waterford, NY, USA), and the suspensions were spread to be 10 μL/cm^2^ on the leaf disks. After Cry toxins suspension were dried at room temperature, two leaf disks were put in each petri dish with 10 larvae of 2nd instar of the genome edited, and wild-type strains were respectively reared on each disk for 2 days. After 2 days, the larvae were moved to non-toxins leaf disks and mortality was recorded at 4 days after feeding initiation.

The median lethal dose (LC_50_) values and the 95% confidence interval were calculated based on Probit Analysis.

### 4.7. cDNA Cloning of BmABCA2 and Construction of Expression Vectors for HEK293T Cells

Total RNA was isolated from midgut tissue of 5th instar larvae of the wild-type silkworm strain, using TRIzol (Thermo Fisher Scientific, Waltham, MA, USA) according to the protocols of manufacturer, and used for cDNA synthesis as a template. The BmABCA2 cDNA was amplified by one-step RT-PCR using PrimeScript High Fidelity RT-PCR Kit (TaKaRa Bio). The amplified cDNA using primers (forward: 5′-CCACCCGGATCCGATATGAGACCTCAGAGAAAAGAAGCC-3′, reverse: 5′- GTCTTTGTAGTCGATCAAGCCTTCCCTTTGATATTTCGT-3′) was cloned into *EcoR*V site of the pcDNA3.1 (Thermo Fisher Scientific, Waltham, MA, USA) using In-Fusion^®^ HD Cloning Kit (TaKaRa Bio, Kusatsu, Japan). The neomycin resistance gene of pcDNA3.1 was replaced by Enhanced green fluorescent protein (EGFP) - *Streptoalloteichus hindustanus* ble (Sh ble) fusion gene by In-fusion cloning method described below. The linearized vector was generated from pcDNA3.1 plasmid as a template by PCR using primers (forward: 5′-GCCCTTGCTCACCATGCGAACGATCCTCATCCTGTC-3′, reverse: 5′- GAGGAGCAGGACTGAGCGGGACTCTGGGGTTCG-3′). The EGFP-ble fusion gene was amplified using primers (forward: 5′-ATGGTGAGCAAGGGCGAGGAG-3′, reverse: 5′- TCAGTCCTGCTCCTCGGCCAC-3′) and cloned into the linearized vector.

### 4.8. Expression of BmABCA2 and BmABCC2 in HEK293T Cells and Cell Swelling Assay with Cry Toxins

HEK293T cells were cultured and transfected, as described previously [5]. The HEK293T cells were cultured on the cover glass in a 6-well plate (Truesline; Nippon Genetics, Tokyo, Japan). Until the HEK293T cells grow up to 70 ~ 80% confluence, expression vectors for BmABCA2 and BmABCC2 were transfected in Opti-MEM^®^ (Thermo Fisher Scientific, Waltham, MA, USA) with polyethylenimine (PEI Max, Polysciences, Warrington, PA, USA) for 2 h incubation at 37 °C. Then, the media were changed to a fresh Dulbecco’s modified Eagle medium (D-MEM) and incubated at 37 °C for 48 h in a CO_2_ incubator. After that, the cover glass was taken out and covered onto Cry toxins solutions in a hole of the 2-hole glass slide. Cry toxins were diluted with PBS (pH 7.4). After 1 h incubation at 37 °C, the cells were observed by phase-contrast and fluorescent microscopy.

## Figures and Tables

**Table 1:** Responses of the knock-out (A2T14) and wild-type strains to Cry toxins.

	*B. mori* Strains	N ^1^	Cry Toxin LC_50_ (ppm, 95%CI) ^2^	Slope ^3^	RR ^4^
2Aa	A2T14	180	>332	-	>9.182
	Wild-type	210	36.155 (26.431–49.518)	2.173	1.000
2Ab	A2T14	180	>4096	-	>9990.244
	Wild-type	270	0.410 (0.302–0.799)	2.114	1.000
1Aa	A2T14	240	1.382 (1.152–1.730)	4.009	1.544
	Wild-type	240	0.895 (0.705–1.128)	2.410	1.000
1Ab	A2T14	210	4.808 (3.818–6.344)	1.994	1.057
	Wild-type	210	4.616 (3.515–6.771)	2.119	1.000
1Ac	A2T14	210	22.078 (17.150–28.664)	2.116	4.212
	Wild-type	210	5.242 (3.579–9.043)	1.746	1.000
1Ca	A2T14	240	0.450 (0.365–0.567)	2.916	0.569
	Wild-type	210	0.791 (0.530–1.627)	1.478	1.000
1Da	A2T14	210	0.836 (0.600–1.301)	1.498	1.101
	Wild-type	210	0.759 (0.596–0.981)	2.497	1.000
1Fa	A2T14	210	57.116 (47.210–73.042)	3.801	2.514
	Wild-type	240	22.719 (17.757–30.446)	1.816	1.000
9Aa	A2T14	180	0.264 (0.183–0.467)	1.492	0.708
	Wild-type	180	0.373 (0.311–0.475)	3.985	1.000

^1^ Number of larvae tested. ^2^ Concentration of toxins killing 50% of larvae and its 95% confidence interval (CI). ^3^ Slope of the concentration-mortality line. ^4^ Resistance ratio (RR) = LC50 of knock-out strain divided by LC50 of the same toxin for wild-type.

## Supplementary Materials

The following are available online at https://www.mdpi.com/2072-6651/12/2/104/s1, Figure S1: Mutations in BmABCA2 in mutant strains produced by genome-editing method using TALENs.
