# Supplementary material for: ATP-Binding Cassette Subfamily a Member 2 Is a Functional Receptor for Bacillus thuringiensis Cry2A Toxins in Bombyx mori, But Not for Cry1A, Cry1C, Cry1D, Cry1F, or Cry9A Toxins
Source: Toxins (Basel). 2020 Feb 6;12(2):104. doi: 10.3390/toxins12020104 (PMC7076765; doi:10.3390/toxins12020104)
Supplement: Supplementary file 1 [file toxins-12-00104-s001.zip › Fig.S1.pdf]

```
WT      GCTGTTCCAATAGTTATGATGAATGCGACGCTGCTGATCGTACAGTTCCTCATTTCGTTCGTTGCCAATATC
        AlaValProIleValMetMetAsnAlaThrLeuLeuIleValGlnPheLeuIleSerPheValAlaAsnIle

A2T01   GCTGTTCCAATAGTTATGATGAATGCGACG-------------------------TAAGCTTTGCCAATATC
        AlaValProIleValMetMetAsnAlaThr                         Stp
A2T03   GCTGTTCCAATAGTTATGATGAATGCGA---------TCGTACAGTTCCTCATTTCGTTCGTTGCCAATATC
        AlaValProIleValMetMetAsnAlaIle         ValGlnPheLeuIleSerPheValAlaAsnIle
A2T06   GCTGTTCCAATAGTTATGATGAATGC-----TGCTGATCGTACAGTTCCTCATTTCGTTCGTTGCCAATATC
        AlaValProIleValMetMetAsnAla     AspAspArgThrValProHisPheValArgAlaGlnTyr
A2T08   GCTGTTCCAATAGTTATGATGAATGCG---CTGCTGATCGTACAGTTCCTCATTTCGTTCGTTGCCAATATC
        AlaValProIleValMetMetAsnAla   LeuLeuIleValGlnPheLeuIleSerPheValAlaAsnIle
A2T11   GCTGTTCCAATAGTTATGATGAATGCG------CTGATCGTACAGTTCCTCATTTCGTTCGTTGCCAATATC
        AlaValProIleValMetMetAsnAla      LeuIleValGlnPheLeuIleSerPheValAlaAsnIle
A2T14   GCTGTTCCAATAGTTATGATGAA----ACGCTGCTGATCGTACAGTTCCTCATTTCGTTCGTTGCCAATATC
        AlaValProIleValMetMetLys    ArgCysStp
```

Figure. S1 Mutations in BmABCA2 in mutant strains produced by genome-editing method using TALENs. The predicted cDNA sequences and their deduced protein sequences from the wild-type (WT) and TALEN mutant strains, showing premature termination of the ABCA2 protein in the mutant strains.
